# Feasibility and impact of three-dimensional (3D) printing technology in simulated teaching of congenital malformations

**DOI:** 10.1186/s12909-024-05506-y

**Published:** 2024-05-05

**Authors:** Qi Gao, Qi Wang, Mingming Li, Chaoxiang lu

**Affiliations:** Department of Neonatal Surgery, Xi’an Children’s Hospital, 69 Xiyuyuanxiang, Xi ’an City, Shaanxi Province 710003 China

**Keywords:** 3D printing, Pediatric surgery, Standardized teaching, Simulation teaching

## Abstract

**Aims:**

This study aimed to investigate the feasibility and effectiveness of utilizing three-dimensional (3D) printing technology in the simulation teaching of congenital malformations.

**Methods:**

We conducted a comparative analysis between an experimental group that received traditional teaching supplemented with 3D printing model demonstrations and hands-on model operation, and a control group that received traditional teaching methods. Various parameters, including classroom interest, classroom interaction, learning enthusiasm, disease awareness, teaching satisfaction, and independent operation confidence, were assessed, along with theoretical and practical tests.

**Results:**

The results showed no significant difference in theoretical test scores between the two groups (91.92 ± 15.04 vs. 89.44 ± 14.89), but the practical test revealed a significantly higher number of qualified trainees in the experimental group compared to the control group (23 vs. 8). In terms of classroom engagement, both groups exhibited similar levels of interest (8.08 ± 1.52 vs. 8.74 ± 0.984), classroom interaction (7.88 ± 1.97 vs. 8.7 ± 1.33), learning enthusiasm (8.81 ± 1.021 vs. 8.52 ± 1.189), and disease awareness (8.58 ± 0.99 vs. 8.58 ± 0.99). However, the experimental group demonstrated significantly higher teaching satisfaction (8.81 ± 1.06 vs. 9.19 ± 0.96) and greater operation confidence (7.67 ± 2.56 vs. 5.5 ± 2.79) than the control group.

**Conclusion:**

3D printing technology can be effectively utilized to create surgical teaching models, enhancing the confidence of standardized training doctors and improving teaching outcomes.

## Introduction

Congenital malformations are the most common pediatric surgical diseases, accounting for approximately 50% of all pediatric surgical cases. They represent a significant component of pediatric surgical education, as surgical intervention is often a crucial treatment approach [1]. Pediatric surgery, however, demands a high level of proficiency from surgeons, and many procedures have steep learning curves, which may lead to severe complications. Thus expediting the training of pediatric surgeons is a formidable challenge. Residents in standardized training programs have limited time for education, making it difficult for them to acquire essential surgical skills, hindering the achievement of educational objectives.

In recent years, 3D printing technology has emerged as a rapid manufacturing technique capable of constructing objects using various materials, including metals and polymers [2–7]. While 3D printing has found applications in medical fields such as spinal surgery, orthopedics, and dentistry, it has primarily employed rigid metallic materials to create specialized objects for therapeutic purposes. However, many congenital malformations involve soft tissue abnormalities, which are not effectively replicated using conventional 3D printing materials.

Congenital anorectal malformation is the most common digestive tract malformation, followed by Meckel’s diverticulum and infantile hypertrophic pyloric stenosis. Because the classification of congenital anorectal malformations is complex and not suitable for residents, the operation of Meckel’s diverticulum is similar to enteroanastomosis and can be trained with existing models, so we chose infantile hypertrophic pyloric stenosis (IHPS) as the training model. IHPS is the most common condition requiring surgery in infancy. Infantile hypertrophic pyloric stenosis is a common disease in pediatric surgery, typically occurring in infants under three months of age, with the diagnosis often made during the newborn period. The procedure is generally considered to be performed by a trained resident. We used 3D printing in combination with molding techniques to create models that simulate congenital malformations. These models can be directly employed for simulation-based teaching and surgical training, enhancing the educational outcomes. In this report, we present our findings on the application of 3D printing technology in pediatric surgical education, specifically for congenital malformations.

## Objects and methods

### Target audience

A total of 53 resident doctors from the grades 2020 and 2021 at our institute were chosen. They were divided into two groups randomly and none of them have received any prior training on pyloromyotomy procedure. Following the principle of voluntary participation, all research subjects provided informed consent and participated anonymously in the study. The Institutional Review Board (IRB) of the Children’s Hospital in Xi’an deemed this study to be exempt from review.

## Model creation

**Fig. 1:**
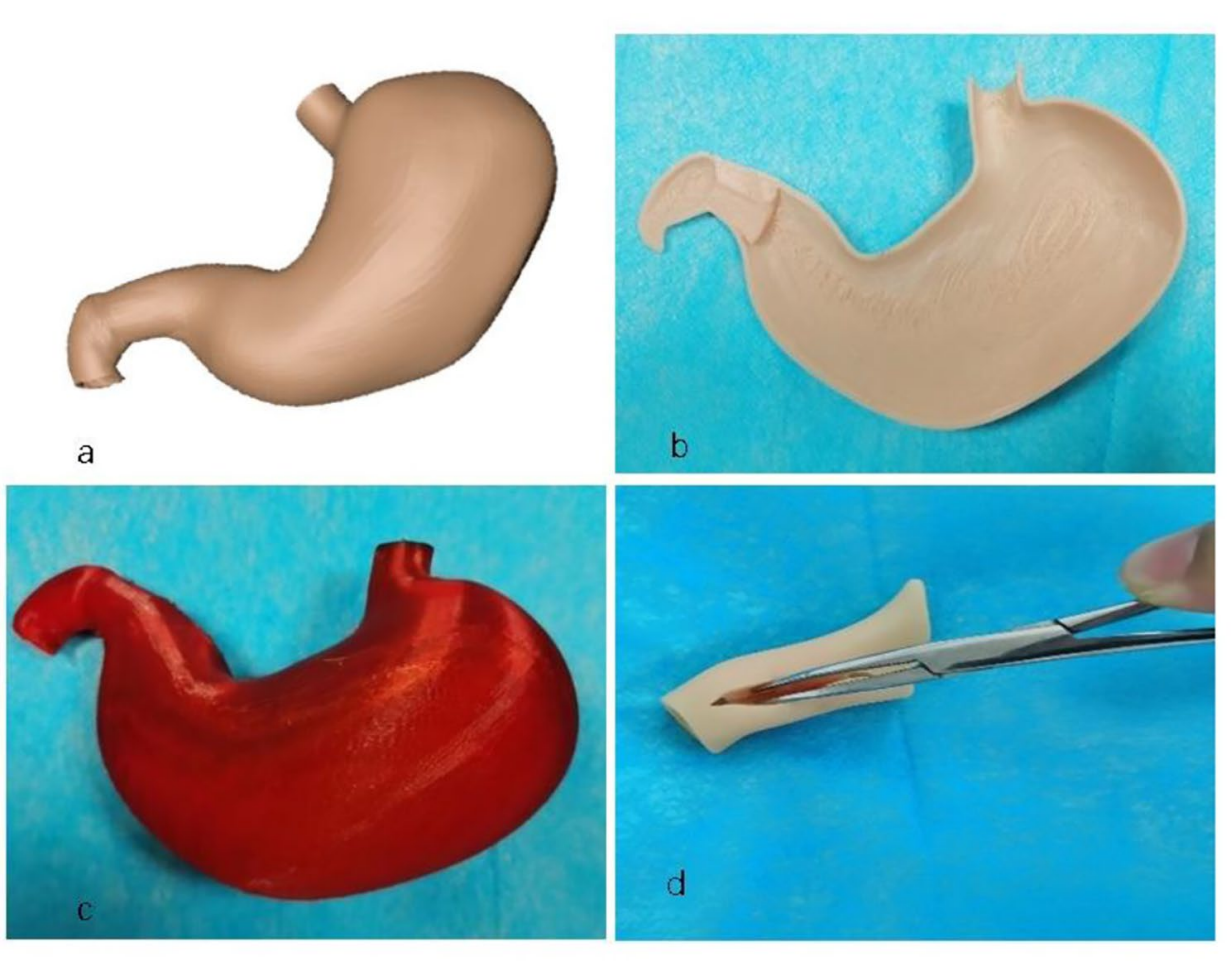
**a.** Constructed pyloric model using software. **b.** Model printed using a 3D printer. **c.** Complete model of infantile hypertrophic pyloric stenosis printed using a flexible material with a 3D printer. **d.** Silicone model used for surgical simulation (separated)

Using infantile hypertrophic pyloric stenosis as an example, a 3D model of this condition was built using specialized software (Fig. 1a). Subsequently, a demonstration model was 3D-printed using a Fused Deposition Modeling(FDM)3D printer (Fig. 1b and c). Through a twice-wrapped technique, a silicone surgical model was realized for surgical simulation (Fig. 1d).

### Grouping method

All individuals willing to participate in the study were first assigned unique codes using Excel, and then random assignment was performed using the random function. Based on the assigned values, participants were divided into the experimental group (27 individuals) and the control group (26 individuals).

The control group received traditional teaching methods, which included multimedia PowerPoint presentations, on-site lectures, and surgical video viewing. In addition to the teaching methods used in the control group, the experimental group received additional demonstrations using 3D printed models and surgical simulations. The duration of the learning sessions was two classes, and the same teacher taught both groups.

### Evaluation method

Immediately after the conclusion of the course, a survey was conducted to assess several aspects, each rated on a scale of 1 to 10, including: class engagement, interactivity, motivation to learn, understanding of the disease, satisfaction with the teaching, and confidence in independent operation. Additionally, students were assessed through a theoretical examination with a maximum score of 100 and an on-site practical assessment, and the assessment scores were recorded.

The junior doctors undergo evaluations of their surgical skills by senior surgeons, which include assessing their theoretical knowledge and practical abilities related to a specific disease. The theoretical knowledge assessment covers the clinical characteristics, physical signs, diagnostic criteria and treatment methods of the disease. The operational grading criteria of pyloromyotomy are as follows: 1.the correct technique for pyloric grasping: Grasp it firmly, ensuring it does not slip.2.Appropriate incision length: The length should be between the stomach and the duodenum, avoiding the vulnerable area of the duodenum. 3. Appropriate incision depth: Cut through the muscle but left the mucosa, no rupture. 4. Complete the procedure within 5 min.

Statistical analysis was performed using SPSS 24.0 statistical software. Descriptive statistics, such as mean ± standard deviation, were used to represent continuous variables. T-tests were conducted for parametric data, while numerical calculations were used for count data. The chi-square test (χ2 test) was employed. A significance level of *P* < 0.05 was considered statistically significant, indicating a significant difference.

## Results

There were no statistically significant differences between the two groups of resident doctors in terms of educational background, gender, age, and years since graduation, as seen in Table 1.

**Table 1:** A Comparison of Basic Characteristics of the Two Groups of Resident Doctors

	Educational Attainment (Bachelor’s/Master’s and above)	Sex(female/male)	Age(Years)	Years Since Undergraduate Graduation (Years)
Control Group(*n* = 26)	20/6	24/2	27.58 ± 2.595	3.38 ± 1.94
Experimental Group(*n* = 27)	22/5	23/4	27.33 ± 1.776	3.26 ± 1.48
χ^2^/T	0.167	-	0.400	0.265
*P*	0.682	0.669	0.691	0.792

The assessment results of the two groups of students indicated no significant difference in theoretical examination scores. However, in terms of practical skills assessment, the experimental group of resident doctors demonstrated a significantly higher number of qualified individuals compared to the control group of resident doctors, as seen in Table 2.

**Table 2:** Comparison of Theoretical and Practical Assessment Scores between the Two Groups of Resident doctors

	Theoretical Assessment Scores	Practical Assessment Scores
Control Group(*n* = 26)	91.92 ± 15.04	8
Experimental Group(*n* = 27)	89.44 ± 14.89	23
T/χ^2^	0.603	16.154
*P*	0.549	**< 0.01**

The theoretical examination scores showed no significant difference between the two groups of resident doctors. However, when it comes to the practical skills assessment, the experimental group of resident doctors performed significantly better than the control group of resident doctors.

Survey questionnaire outcomes showed a marginal disparity between the two cohorts of resident doctors concerning classroom engagement, interactive dynamics, learning enthusiasm, disease awareness, and instructional satisfaction. Notably, the experimental group exhibits a pronounced elevation in operational self-assurance compared to their counterparts in the control group.(Table 3).

**Table 3:** Presents a comparative analysis of survey responses from the two groups of Resident Doctors

	Classroom Enjoyment	Interactive Classroom Dynamics	Learning Enthusiasm	Disease Awareness	Teaching Satisfaction	Confidence in Independent Operations
Control Group(*n* = 26)	8.08 ± 1.52	7.88 ± 1.97	8.81 ± 1.021	8.58 ± 0.99	8.81 ± 1.06	5.5 ± 2.79
Experimental Group(*n* = 27)	8.74 ± 0.984	8.7 ± 1.33	8.52 ± 1.189	9.81 ± 1.11	9.19 ± 0.96	7.67 ± 2.56
T	-1.893	-1.785	0.949	-0.823	-1.359	-2.950
*P*	0.064	0.080	0.347	0.414	0.180	**0.005**

## Discussion

Clinical practical skills are core competencies for resident doctors and are particularly crucial for surgical residents’ training in surgical skills. However, the training of surgical skills for resident doctors has consistently been a weak point in the majority of standardized training programs, calling for urgent improvement [8]. A key issue hindering the development of surgical skills training is the lack of a safe, effective, convenient and affordable surgical simulation training device.

In recent years, 3D printing technology has emerged as a rapid production technique that enables the construction of objects using materials such as metals and polymers. Utilizing 3D printing technology can enhance students’ understanding of congenital deformities [9], as well as improve teaching and surgical proficiency [10, 11]. Previous 3D modeling often relied on direct modeling from 3D imaging data derived from CT or MRI, which had its advantages in terms of simplicity and accuracy in modeling the actual patient anatomy [12]. However, it was challenging to construct models of human soft tissues using imaging data, which predominantly focused on orthopedics [13]. This study attempted to simulate the human digestive system using software modeling combined with 3D printing technology and silicone gel molding, achieving encouraging results and surpassing the limitations of previous studies. With the abundance of online learning resources available, tutorials can be conveniently accessed through the internet. Once models are established, they can be saved and adjusted according to teaching needs, such as altering model specifications and dimensions.

The field of pediatric surgery encompasses numerous congenital structural deformities, including intestinal obstruction, intestinal stenosis, choledochal cysts, annular pancreas, esophageal atresia with tracheoesophageal fistula, and others. Although these deformities have a low incidence rate, they are common diseases in pediatric surgery and an integral part of resident physician training programs [14]. The scarcity of industrial-scale production often hinders the availability of actual molds for these rare models, making their procurement challenging. Moreover, utilizing animal models for training incurs high time and economic costs, with a low success rate in replicating deformities, making it impractical. Directly allowing students to perform surgeries on patients, especially newborns, raises concerns over high surgical risks and ethical dilemmas. These factors contribute to the difficulty in pediatric surgical resident training, making the judicious use of 3D printing technology essential in improving teaching outcomes [1].

Williams reported 3D-printed stomach model for simulated laparoscopic pyloromyotomy is a useful training tool for learners to improve laparoscopic skills [15]. In our study, we recruited more residents whose work and learning experiences were very similar for better comparison. In our study, we recruited more physicians whose work and learning experiences were very similar for better comparison. We came to similar conclusions.

Currently, many training centers for resident doctors utilize laparoscopic surgery to treat this condition. More studies were incorporated and the learning curve for hypertrophic pyloric stenosis in infants was between 10 and 35, in which most is over 15 [16–20]. During resident rotations in neonatal surgery for 4 months, they rarely have access to more than 15 patients with IHPS, which also prevents them from achieving proficiency in the technique. In the current situation in our region, it is difficult for junior doctors to complete laparoscopic pyloromyotomy, so we only conducted tests using open surgery. We believe that this is more suitable for testing the skills of resident doctors. However, due to the early onset of the disease, which may occur in newborns, the delicate nature of the tissues in these infants poses a high risk for resident doctors directly participating in such surgical procedures. This not only compromises patient safety but also goes against medical ethics.

By incorporating software modeling and 3D printing technology into the teaching of infantile hypertrophic pyloric stenosis, the author has significantly improved students’ confidence in surgical procedures. A survey revealed no significant differences between the two groups of resident doctors in terms of classroom engagement, classroom interaction, learning motivation, disease recognition, and teaching satisfaction. This could be attributed to the fact that simulation modeling in clinical teaching is not a novel concept, and the use of models in teaching various diseases has diminished the novelty of 3D printing models. Additionally, it may be influenced by the teaching proficiency of the instructors. In the theoretical and practical stages, the 3D printing models did not noticeably improve theoretical scores, but in the practical stage, the control group of resident doctors demonstrated improved accuracy, proficiency, and overall success rate in surgical procedures. 3D printed models still have relatively high costs. Further large-scale implementation may lead to cost reduction. By adding training methods for 3D printed models, it enhances the resident doctors’ confidence in their skills and improves the success rate of the operation.

In the future, as material technology improves, 3D printed models will simplify the way these models are made. So we can print out more realistic surgical simulation equipment. It can be used both to train young physicians and as a pre-operative simulation for complex surgeries.

### Limitations

1. Single-center study: This study was conducted at a single center and requires further multicenter research for validation.
2. Discrepancy in tactile feedback: There exists some disparity between 3D printed intestinal models and actual organs in terms of tactile sensation and other factors.
3. Modeling technique and proficiency: The quality of the models depends on the mastery of modeling techniques. Learning the skills for 3D printing technology requires a certain level of computer literacy.
4. The control group didn’t have any type of hands-on learning, so it seems that they were at a disadvantage.

## Conclusion

The utilization of software modeling and 3D printing for constructing teaching models of congenital deformities is feasible. It can enhance the confidence and performance of surgical resident doctors, improve teaching outcomes, and address the challenges in practical training for rare deformities in pediatric surgery. This approach is worthy of further promotion.

## Data Availability

The datasets generated and/or analyzed during the current study are not publicly available due to the confidentiality and sensitivity of the survey data and the stated terms with the survey respondents, but they can be made available from the Corresponding Author on reasonable requests. No datasets were generated or analysed during the current study.

## Declarations

### Finding

Residential training base construction project provided by Xi ‘an Health Commission (No 2022zp09).

### Clinical trial registration (if any)

Not involved.
